# Trends and outcomes of fertility preservation in patients presenting with cancer during pregnancy or postpartum—a longitudinal observational cohort study

**DOI:** 10.3389/fendo.2026.1771764

**Published:** 2026-02-16

**Authors:** Hanna P. Nilsson, Riikka Bornhede, Anna L. V. Johansson, Kenny A. Rodriguez-Wallberg

**Affiliations:** 1 Department of Oncology-Pathology, Laboratory of Translational Fertility Preservation, Karolinska Institutet, Stockholm, Sweden; 2 Department of Women’s Health, Division of Obstetrics, Karolinska University Hospital, Huddinge, Sweden; 3 Department of Medical Epidemiology and Biostatistics, Karolinska Institutet, Stockholm, Sweden; 4 Department of Reproductive Medicine, Division of Gynecology and Reproduction,Karolinska University Hospital, Stockholm, Sweden

**Keywords:** cancer in pregnancy, fertility preservation, infertility, oncofertility, pregnancy associated cancer, young adult cancer survivors, female cancer, cancer postpartum

## Abstract

**Objective:**

The aim of this study was to assess the trends and outcomes of fertility preservation (FP) in women referred for FP counseling and presenting with pregnancy-associated cancer (PAC).

**Method:**

This is a prospective cohort study of all patients referred for FP counseling between 2001 and 2024 to the FP program of Karolinska University Hospital, Sweden. Baseline data, age, parity, disease stage, treatment characteristics, and FP methods were retrieved from clinical registries.

**Results:**

A total of 50 women with cancer diagnosed coincidentally with pregnancy (79%) or up to 1 year after delivery (21%) were referred for FP counseling. Among them, 30 women chose to proceed with FP; 10 by either hormonal stimulation to freeze eggs/embryos or ovarian tissue cryopreservation (OTC) after abortion/miscarriage, 10 by OTC at delivery, and 9 were planned for FP postpartum. The most common cancers were breast cancer (*N* = 31, 62%), cervical cancer (*N* = 6, 12%), and lymphoma (*N* = 5, 10%). Most women diagnosed with cancer in the first trimester either terminated the pregnancy or had a miscarriage (76%). All patients diagnosed in the second and third trimesters delivered through cesarian section (*N* = 14), scheduled from week 31 and onwards. All patients diagnosed in the third trimester started cancer treatment postpartum. In the FP group, 57% cryopreserved ovarian tissue postpartum or post-abortion and 43% underwent ovarian stimulation for oocyte/embryo cryopreservation prior to chemotherapy initiation. Four women proceed to FP after chemotherapy, three by ovarian tissue freezing and one through attempted, unsuccessful, hormonal stimulation. After a mean follow-up of 9.9 years, 45 patients were alive. The proportion of women having previous children at diagnosis was the same among the FP and no-FP groups. At the end of follow-up, the percentage of nulliparous women was 20% in the no-FP group and 13% in the FP group.

**Conclusions:**

Our observations underscore the need to ensure good multidisciplinary communication to inform patients presenting with PAC on the future risk for infertility and on the available FP procedures. As FP has to be applied when the patients are not pregnant, these measures can be planned in connection with a cesarean section, or after completion of cancer treatment. Current guidelines for FP lack specific recommendations for women with PAC, and specialized PAC guidelines also lack specific information on FP. Clinical Trial Registration: ClinicalTrials.gov, identifier NTC04602962.

## 1 Introduction

Pregnancy-associated cancers (PACs), i.e., cancer diagnosed during pregnancy or within 1 year of delivery, are rare, but increasing in incidence, occurring approximately once in every thousand pregnancies (1–3). The increased incidence can at least partly be linked to an older population giving birth (4). Currently, one-fourth of all PACs are diagnosed during pregnancy (PrC) and the remaining cases are diagnosed in the postpartum year (PPC) (3). In population-based settings, the most common malignancies presenting during pregnancy are melanoma, breast cancer, cervical cancer, lymphoma, and leukemia (4, 5).

Recent treatment recommendations on PrC suggest adhering to standard cancer treatment, maintaining the pregnancy, and avoiding prematurity as far as possible. Vaginal delivery is only discouraged in case of certain gynecological cancers; in all other cases, cesarean section should only be planned on obstetric indication (6, 7). These recommendations are based on an increasing number of studies showing reassuring obstetric and health-related outcomes in the children born after exposure to treatment for cancer *in utero* (8–10). However, gestational age at cancer diagnosis remains a main determinant in planning treatment for cancer during pregnancy. Chemotherapy is commonly not recommended until the second or third trimester, as chemotherapy in the first weeks of pregnancy has been shown to induce abortion and negatively affect organogenesis, potentially causing severe congenital malformations and cognitive impairment in the fetus (11). In advanced-stage disease, women before gestational week 22 might also be counseled to terminate the pregnancy in order for the treatment to proceed immediately. During the later stages of pregnancy, most chemotherapeutic drugs can be utilized with relative safety, while irradiation is usually not recommended until after delivery unless there is no other option (12).

While all young women diagnosed with cancer should be offered fertility counseling, fertility preservation (FP) measures are not mentioned in the PrC/PPC guidelines and FP is thus indicated primarily based on the estimated gonadotoxicity of the cancer treatment (13, 14). Established FP options include cryopreservation of ovarian tissue, oocytes, or embryos, as well as strategies for fertility-sparing surgery (13, 14). All available FP measures are achievable at the earliest at delivery or after termination of the pregnancy. While the success rate of FP is age-dependent and FP measures are most effective prior to gonadotoxic exposure, if infertility is not immediate, in many cases FP can also be implemented after completed cancer treatment to counteract the effects of treatment-induced reproductive ageing (15).

The current data on FP choices and adherence to recommendations in women that are pregnant or postpartum at the time of cancer diagnosis are very limited. The available studies on the psychosocial aspect of PAC highlight an increased anxiety, as well as a sense of conflict and guilt surrounding healthcare decisions impacting the future wellbeing of both mother and child (16–18). Long-term psychological distress has been related to not receiving FP, being advised to terminate the pregnancy, worrying about preterm delivery, cesarean delivery, and cancer recurrence (19).

With this longitudinal cohort study, we wish to shed light on the trends and outcomes of FP in patients with PAC as fertility after cancer remains a concern not only in women with no children at diagnosis but also in most women even with prior children (20, 21).

## 2 Material and methods

The study cohort included all women with PAC, here defined as a cancer diagnosed or treated coincidentally with a pregnancy and up to 1 year postpartum, who were referred for FP counseling at the Karolinska University Hospital, Section of Reproductive Medicine, in Stockholm, Sweden, between 1 January 2001 and 31 December 2024. Last follow-up was March 2025. Restrictions limiting access to the tax-funded FP measures provided at the clinic include age ≥40 years and more than one previous child. Data on referral, clinical characteristics and utilization of cryopreserved oocytes and tissues have been collected prospectively.

Ethical approval was granted by the Ethical Review Board of Karolinska University Hospital (Dnr 427/03) and the Regional Ethics Committee of Stockholm (Dnr 2011/1158-31/2, 2014/470-32, 2016/2530-32, and 2018/2255-32). Patient consent was obtained at the time of FP counseling; orally until 2008 and thereafter in writing. Participation in the study did not affect the clinical treatment offered.

The clinical recommendations for FP have changed over time, and the women have been predominantly counseled towards clinically established methods. Women planned for chemotherapy with a short delay are prioritized for emergency appointments. When the pregnancy was advanced at the time of referral, the scheduled appointments were informational, as FP measures can be done at the earliest at time of delivery or after termination of the pregnancy. FP counseling included information on both available FP measures and alternatives to becoming a parent, such as egg donation or adoption. In case the patients opted not to undergo FP at the time of referral, the possibilities and limitations of undergoing FP at a later stage were discussed. When deemed necessary, ovarian reserve was evaluated through serum concentration of anti-Mullerian hormone (AMH) and/or antral follicle count (AFC). Further details on FP counseling have been previously reported (22). Data on FP procedures, cancer recurrence, and clinical variables were extracted from the prospectively updated electronic treatment databases (LinnéFiler^®^ and TakeCare^®^). Demographic and clinical characteristics of the patients are presented descriptively as numbers, proportions, and means, with mean differences evaluated through a two-sided *t*-test with a significance level of 0.05 and percentages through a chi-square test. All statistical analyses were executed in Excel.

## 3 Results

### 3.1 Cohort demographics

In total, 50 patients with PAC were referred for fertility counseling at Karolinska University Hospital, Section of Reproductive Medicine, between 2001 and 2024. A majority of the patients were referred for counseling after 2011 (84%) (**Table 1**), peaking with 11 patients referred in 2017 whereafter the number of referrals declined. All patients were adult at the time of referral (mean age, 32 years; range, 22–41). Mean BMI was 25.5 (**Table 1**). Half (50%) of the patients had previous children (**Table 2**) and all but one were in an ongoing relationship at diagnosis (**Table 1**). Mean AMH was 2.0 μg/L, ranging from 0.2 to 12.5. Among the patients undergoing a vaginal ultrasound, mean AFC was 15.2, ranging from 6 to 35. In the cohort, 30 patients chose to proceed with FP after referral. No significant differences were observed when comparing the groups choosing to proceed with FP and those that did not (FP vs. No FP: Age, *p* = 0.20; BMI, *p* = 0.19; AFC, *p* = 0.74; AMH, *p* = 0.47; Parity, *p* = 1.00).

**Table 1:** Cohort characteristics.

Characteristics	Total, *N* (%)	FP, *N* (%)	No FP, *N* (%)
Total	50 (100%)	30 (100%)	20 (100%)
Year of diagnosis			
2001–2010	8 (16%)	7 (23%)	1 (5%)
2011–2020	32 (64%)	18 (57%)	14 (70%)
2021–2024	10 (20%)	5 (10%)	5 (25%)
	Total, mean ± SD (range)	FP, mean ±SD (range)	No FP, mean ± SD (range)
Age (years)*	32 ± 3.9 (22–41)	32 ± 3.3 (22–39)	33 ± 4.2 (26–41)
Years follow up post FP		11.1 (3–24)	–
BMI (kg/m^2^)* Missing = 9	25.5 ± 5.0 (19.6–39.2)	26.3 ± 5.4 (19.6–39.2)	24.2 ± 4.1 (18.0–28.7)
AFC (*n*)* Missing = 27	15.2 ± 9.0 (6–35)	14.6 ± 8.4 (4–35)	16.1 ± 10.7 (6–38)
AMH (µg/L)* Missing = 15	2.0 ± 2.4 (0.2–12.5)	2.1 ± 3.0 (0.2–12.5)	1.6 ± 1.5 (0.53–5.9)
Partner at diagnosis Missing = 4	Total, *N* (%)	FP, *N* (%)	No FP, *N* (%)
Yes	45 (98%)	26 (100%)	19 (95%)
No	1 (2%)	0 (0%)	1 (5%)
Diagnosis			
Breast cancer	31 (62%)	18 (60%)	13 (65%)
Cervical cancer	6 (12%)	3 (10%)	3 (15%)
Lymphoma	5 (10%)	4 (13%)	1 (5%)
Other	8 (16%)	5 (17%)	3 (15%)
Gynecological surgery			
Trachelectomy	3 (6%)	2 (7%)	1 (5%)
Hysterectomy	2 (4%)	1 (3%)	1 (5%)
No surgery	45 (90%)	27 (90%)	18 (90%)
Previous chemotherapy	6 (12%)	4 (13%)	2 (10%)
Chemo Missing = 12			
Yes	34 (89%)	22 (92%)	12 (86%)
No	4 (11%)	2 (8%)	2 (14%)

**Table 2:** Pregnancy status.

Characteristics	Total *N* (%)	FP *N* (%)	No FP *N* (%)
Total	50 (100%)	30 (100%)	20 (100%)
Pregnancy status at cancer diagnosis Missing = 3			
Postpartum	10 (19%)	7 (24%)	3 (17%)
At abortion	2 (4%)	2 (7%)	0 (0%)
First trimester	17 (36%)	9 (31%)	8 (44%)
Second trimester	6 (11%)	2 (7%)	4 (22%)
Third trimester	8 (21)	7 (24%)	1 (6%)
During cancer treatment	4 (9%)	2 (7%)	2 (11%)
Pregnancy status at FP intervention/consultation Missing = 3			
Postpartum	23 (46%)	18 (60%)	5 (29%)
Post abortion	16 (32%)	10 (33%)	6 (35%)
First trimester	4 (8%)	0 (0%)	4 (24%)
Second trimester	2 (4%)	0 (0%)	2 (12%)
Third trimester	1 (2%)	0 (0%)	1 (6%)
Pre-pregnancy	1 (2%)	1 (3%)	0 (0%)
Pregnancy terminated post diagnosis (Spontaneous and induced abortions)			
Pre-pregnancy at diagnosis (*n* = 4)	1 (25%)	0 (0%)	1 (50%)
First trimester (*n* = 17)	13 (76%)	8 (89%)	5 (63%)
Second trimester (*n* = 5)	0 (0%)	0 (0%)	0 (0%)
Third trimester (*n* = 10)	0 (0%)	0 (0%)	0 (0%)
Parity at diagnosis			
0	25 (50%)	15 (50%)	10 (50%)
1	25 (50%)	15 (50%)	10 (50%)
≥2	0 (0%)	0 (0%)	0 (0%)
Parity at FP consult/treatment			
0	16 (32%)	6 (20%)	10 (50%)
1	34 (62%)	23 (77%)	8 (40%)
≥2	3 (6%)	1 (3%)	2 (10%)
Documented parity at follow-up			
0	8 (16%)	4 (13%)	4 (20%)
1	29 (58%)	18 (60%)	11 (55%)
≥2	13 (26%)	8 (27%)	5 (25%)

### 3.2 Cancer and cancer treatment

The majority of women presented with breast cancer (*N* = 31, 62%), six had cervical cancer (12%), five had lymphoma (10%), and eight had other types of cancer (16%). At time of study inclusion, most patients were newly diagnosed and referred for acute fertility counseling prior to their planned chemotherapy treatment (89%). Only a minor proportion of women were planned for gynecological surgeries including trachelectomy (6%) and hysterectomy (4%) (**Table 1**). Patients were referred to the reproductive medicine clinic presenting with cancer stages 0–III. Among the patients with breast cancer (*N* = 29, 2 missing), 52% were stage III (*N* = 15) and 41% were either stage II or stage II–III (*N* = 12).

Six patients had received chemotherapy at some point prior to FP referral. Three patients had received previous chemotherapy due to Hodgkin’s lymphoma; among them, one was now referred for FP counseling due to a pregnancy coinciding with a relapse, and two due to primary breast cancer diagnosed during pregnancy. Three patients referred for fertility counseling postpartum had been treated for breast cancer with chemotherapy during their pregnancies. All patients diagnosed in the third trimester started cancer treatment after delivery.

### 3.3 Pregnancy status

Most patients were referred to FP counseling presenting with cancer occurring coincidental with pregnancy (*N* = 37) (79%). In this group, two cases of cervical cancer were discovered at abortion, and in four cases, the women found out about their pregnancies during the cancer treatment. The remaining women (*N* = 10) presented with cancer during the first postpartum year (21%).

A majority of the women diagnosed with cancer in the first trimester either terminated the pregnancy or had a spontaneous abortion (76%) (**Table 2**). In women without FP, a higher percentage chose to proceed with the pregnancy if diagnosed in the first or second trimester compared to the FP group (63 vs. 27%, *p* = 0.093). In this group of women, six had previous children, and among them, no one proceeded with FP at delivery or postpartum.

All patients diagnosed in the second and third trimesters (*N* = 14) delivered through cesarian section, which were scheduled from week 31 and onwards.

All pregnancies proceeding past the first trimester ended in live birth with the exception of a spontaneous abortion in week 15 occurring 2 weeks after a trachelectomy.

### 3.4 Fertility preservation

Most patients undergoing FP measures did an ovarian biopsy (57%) and proceeded to cryopreserve ovarian tissue and, in some cases, oocytes. In the case of planned cesarean sections, the ovarian biopsy was often retrieved during delivery.

Ovarian stimulation for oocyte retrieval was done in 43% of the patients, whereafter oocytes and/or embryos were cryopreserved. One woman treated with trachelectomy for cervical cancer and planned for FP through embryo freezing 4 months later decided to attempt pregnancy and underwent embryo transfer with a successful pregnancy delivered by cesarean section. In two women, ovarian stimulations did not result in any mature oocytes, and in one case, only a single oocyte matured, whereupon fertilization failed (**Table 3**). Since 2019, all patients have undergone FP by ovarian tissue cryopreservation.

**Table 3:** Fertility preservation.

Characteristics	Total FP, *N* (%)	2001–2010 *N* (%)	2011–2020 *N* (%)	2021–2024 *N* (%)
Total	30 (100%)	7 (100%)	18 (100%)	5 (100%)
Tissue biopsy *N* (%)	16 (57%)	6 (86%)	5 (33%)	5 (100%)
Stimulation *N* (%)	14 (43%)	1 (14%)	13 (67%)	0 (0%)
Ovarian tissue cryopreservation	16 (53%)	6 (86%)	4 (28%)	5 (100%)
Oocyte cryopreservation	12 (40%)	4 (57%)	7 (39%)	1 (20%)
Embryo cryopreservation	9 (30%)	0 (0%)	9 (50%)	0 (0%)
Failed FP	3 (10%)	1 (14%)	2 (11%)	0 (0%)
Days to FP Missing *n* = 3	Total FP, mean ± SD (range)	2001–2010, mean ± SD (range)	2011–2020, mean ± SD (range)	2021–2024, mean ± SD (range)
All FP	14 ± 20 (0–75)	23 ± 22 (4–57)	15 ± 22(0–75)	4 ± 3 (1–7)
Tissue cryopreservation	15 ± 17 (1–57)	23 ± 22 (4–57)	17 ± 16 (1–43)	4 ± 3 (1–7)
Stimulation	14 ± 25 (0–75)	–	14 ± 25 (0–75)	–

All patients received fertility treatment within a span of 0–75 days from referral, with emergency FP prioritized for the women being planned for chemotherapy treatment with a short delay. Thus, most received treatment within a week, but the mean lead time was 14 days (**Table 3**). Mean time from referral to ovarian tissue cryopreservation has been reduced in recent years [mean days to treatment 2001–2020 vs. 2021–2024 (19.7 vs. 3.6, *p* = 0.046); mean days to treatment 2011–2020 vs. 2021–2024 (16.7 vs. 3.6, *p* = 0.047)].

### 3.5 Follow-up

Mean age at follow-up was 42.3 years, and the mean time of follow-up was 9.9 years (range, 0–24 years). At the end of follow-up, 45 patients were still alive (90%). There were no cases of maternal or neonatal death at delivery and one case of maternal death in the 1-year postpartum period.

Four deaths occurred between 1 and 3 years postpartum.

**Figure 1:**
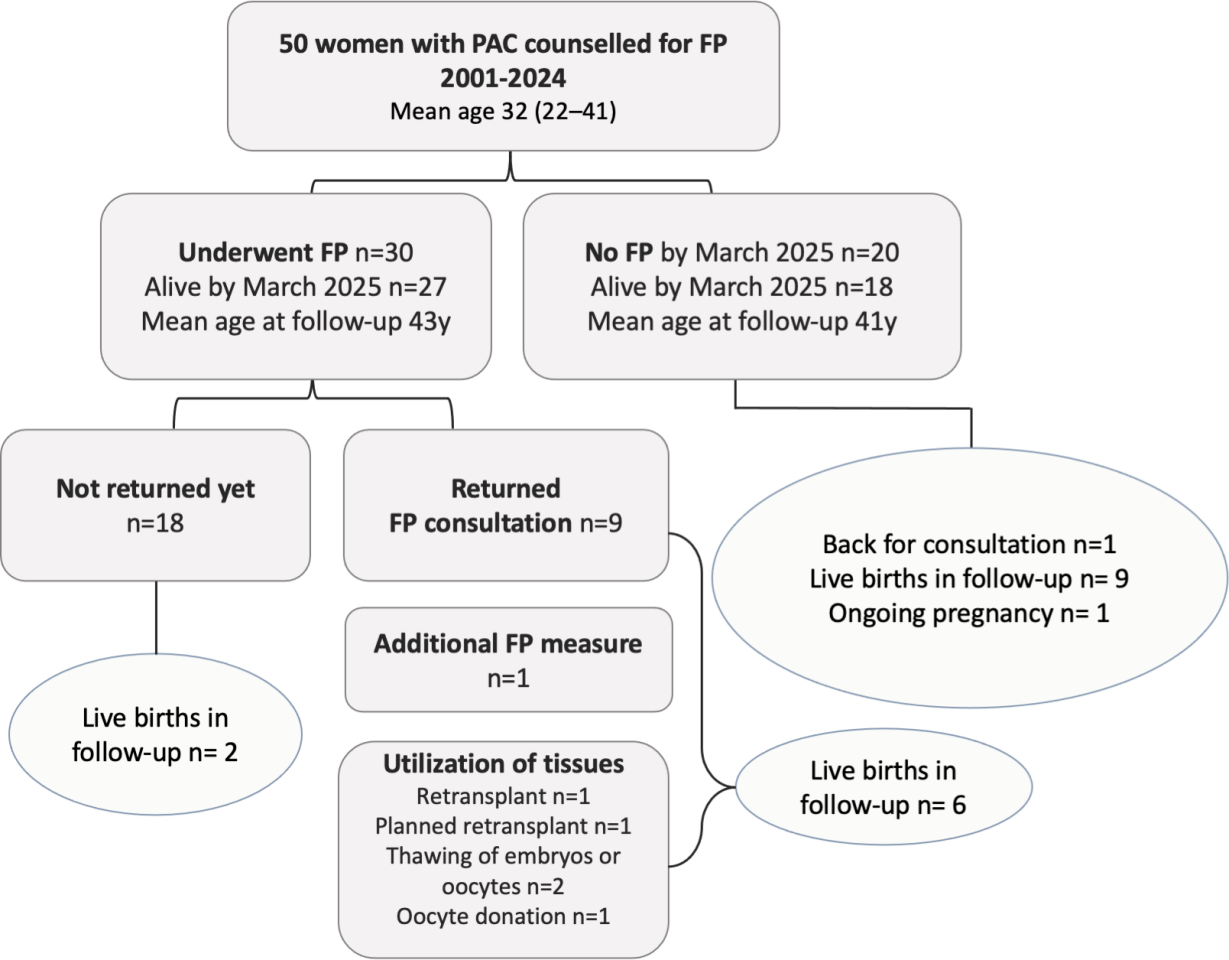
Cohort follow-up.

In the no-FP group (*n* = 20), nine live births were registered post consultation, eight from pregnancies ongoing at cancer diagnosis. One pregnancy was still ongoing at the time of data collection (**Figure 1**). The proportion of nulliparous women decreased from 50% at diagnosis to 20% at the end of follow-up. The proportion of multiparous women increased from 0% to 15% (**Table 2**). One patient returned for further fertility consultations but was not clinically indicated to proceed with FP (**Figure 1**).

In the FP group (*n* = 30), eight live births were registered since FP (**Figure 1**). The proportion of nulliparous women in the cohort decreased from 50% at diagnosis to 13% at the end of follow-up. The proportion of multiparous women increased from 0% to 27% (**Table 2**). Nine women (30%) have returned for further consultation. Among them, one woman did additional stimulation for oocyte cryopreservation. One woman successfully retransplanted ovarian tissue, and one was scheduled for ovarian tissue transplantation in 2025. One woman gave birth through oocyte donation. Two women thawed their frozen oocytes but without resulting pregnancies. Four women became spontaneously pregnant. Among the women not returning for follow-up, two spontaneous pregnancies are recorded (**Figure 1**).

## 4 Discussion

Several studies show that PAC presents an increased risk for the mother and baby including increased risk for preterm birth, cesarean section, small for gestational age babies, venous thromboembolism, and maternal death (23–25).

In our cohort of women with PAC receiving fertility counseling, the overall 90% survival rate is similar to or slightly higher than the survival estimates for breast cancer and cervical cancer recently published from our full FP cohort (26, 27). Most pregnancies coinciding with a first-trimester cancer diagnosis were terminated (76%). Most women that chose to maintain their pregnancy delivered by planned cesarian section, often preterm. In several cases, an iatrogenic cesarian section was scheduled as early as gestational week 31 to allow chemotherapy to start postpartum. This corresponds to the previous reports of increased risk for cesarean section and preterm births among women with PAC (23). Preterm birth in patients with PrC has been linked to adverse outcomes in the children born (28, 29).

The planned cancer treatment was most often initiated after the end of pregnancy, suggesting the occurrence of a post-diagnosis treatment delay that should be further investigated, as each 4-week treatment delay has been linked to increased cancer mortality (30).

These observations underscore the need to improve adherence to the current PAC guidelines and, when necessary, ensure good multidisciplinary communication to allow patients with high risk for infertility to know about FP methods and to have the chance to undergo FP without treatment delay. A good example on how to implement this could be ovarian tissue cryopreservation conducted when there is a planned cesarean section, or a scheduled FP follow-up postpartum to assess fertility potential.

Although specific recommendations on how to approach FP discussions with women diagnosed with PAC are still lacking, medical advances have changed the FP treatment recommendations for patients with cancer in general, and this may be reflected in the observed cohort. During the years 2011–2020, we saw a substantial increase in the number of women presenting with PAC undergoing ovarian stimulation, possibly echoing both the implementation of random-start protocols, aimed at shortening the delay in retrieving oocytes, as well as the implementation of oocyte cryopreservation at our center in 2009, where the successful use of vitrification techniques worldwide ensured approval for oocyte freezing as a clinical method for FP in oncology patients from 2013 (31, 32). Since 2019, we have not performed further ovarian stimulations in our PrC cohort, as ovarian tissue cryopreservation became clinically established and allows FP without delay of treatment initiation (22, 33). Trends in FP choices among patients with PAC mimic the general treatment choices observed in the full cohort of adult women with partners undergoing FP in Sweden and suggest that the FP treatment choice is not notably affected by pregnancy (22).

The modest size of our cohort might reflect that most patients with PAC are not referred for FP or specialized fertility consultations in connection with their cancer diagnosis. The limited referrals are further underscored when considering that large meta-analyses have indicated that only one-fourth of all PAC are diagnosed during pregnancy and the remaining cases are diagnosed in the postpartum period (3). In our cohort, these proportions are reversed (PrC 79% vs. PPC 21%), suggesting an inequality where acute fertility counseling is intuitively offered to patients with PrC, but where patients with PPC do not receive the same opportunities for qualified fertility care acknowledging their PAC status.

The observed peak in PAC FP referrals observed in our cohort between 2013 and 2018, followed by a subsequent decline, may reflect an increase in clinical awareness of FP as a viable option for patients with cancer, followed by the implementation of clinical PrC/PPC guidelines where the perceived need for FP is reduced by solid data reassuring clinicians and patients that cancer treatment can be pursued without compromising maternal or fetal outcomes. As the current guidelines on PrC/PPC encourages maintaining the pregnancy to term, there is little mention of FP as an option (8–10), but even so, the general guidelines on FP do recommend that all patients with cancer of reproductive age should be offered a fertility counseling as soon as possible after diagnosis (13, 14). We suggest that this need for specialized fertility care should be especially acknowledged in patients with PAC in order to allow informed decisions on fertility, inform them of the available FP options, and, when possible, alleviate fears with regard to future childbirths.

At the end of follow-up, 16% of the women in the cohort remained nulliparous. This proportion is comparable to the 13.5% nulliparity rate observed among 45-year-old women in the general Swedish population (34). However, given that all participants in the study cohort had established partnerships and had previously achieved pregnancy, a higher rate of parity than in the general population would be expected. The observed parity distribution could therefore suggest that reproductive outcomes in this cohort were influenced by their cancer diagnosis.

The strengths of this study lie in its long clinical follow-up of all referrals within a rare but exposed patient group. This adds valuable clinical data to the very limited literature available on fertility trends and outcomes in women with PACs. The mean follow-up time was 10 years, and most women in the cohort are now past, or close to, the age limit for further assisted reproduction. Also, this is one of the first Swedish cohorts including outcomes of first-trimester pregnancies. In previous large Swedish register studies on PAC, it has not been possible to identify the cancers terminated early in pregnancy, as only pregnancies lasting beyond gestational week 22 are recorded in the Swedish Medical Birth Register.

The size of the cohort limits the interpretational value of the results as the observed outcomes are not powered to show significant differences. With regard to PAC in general, the cohort has inclusion bias as only women referred for, or actively requesting, fertility counseling were included. For example, malignant melanoma, which is the most common PAC, is not represented, as its surgical treatment is generally not expected to affect fertility.

Notably, a limitation for access to tax-funded FP in Region Stockholm is more than one previous child. While this is not contra-indicative for fertility counseling, it markedly reduces referrals of women multiparous at diagnosis. This bias is reflected in our cohort where the number of women multiparous at diagnosis was 0%, and the number of children per person at the end of follow-up is only 1.12 compared to the total birth rate in Sweden of 1.45 children/women (34). Also, 35% of those choosing not to proceed with FP were women with previous children and ongoing pregnancies at consultation. Among them, no one returned for FP follow-up after giving birth. It is possible that this decision was affected not only by a decrease in pregnancy wish, but also by no longer being eligible for tax-funded FP treatment. While most patients are followed past age 40, the follow-up might lack data on spontaneous pregnancies or privately financed ART.

## 5 Conclusions

Over the years, FP choices and recommendations in patients with PAC have followed the same trends as other FP patient groups. Our observations highlight the rapid developments in treating PAC, and the impact on when, and to whom, fertility consultations are offered. The study underscores a persisting tendency to end pregnancies in patients with PrC preterm and to delay cancer treatment initiation until the postpartum period, indicating a slow implementation of the international PrC guidelines. Furthermore, both the low rate of postpartum cancers and multiparous women referred to fertility counseling highlight a potential gap in survivorship care. These observations underscore the need for structured fertility follow-up in patients with PAC. A possibility could be to routinely integrate reproductive medicine expertise within the PAC multidisciplinary care team, to support informed decision-making, facilitate timely access to FP, and alleviate the documented concerns regarding fertility and child health in this population.

## Data Availability

The datasets presented in this article are not readily available because of the sensitive nature of the clinical data. Requests to access the datasets should be directed to kenny.rodriguez-wallberg@ki.se.
